# Draft Genome Sequence of *Shewanella* sp. Strain ISO12, a Candidate Probiotic Isolated from the Intestine of *Fundulus heteroclitus*

**DOI:** 10.1128/MRA.00399-20

**Published:** 2020-05-28

**Authors:** Harold J. Schreier

**Affiliations:** a Department of Marine Biotechnology, Institute of Marine and Environmental Technology, University of Maryland Baltimore County, Baltimore, Maryland, USA; b Department of Biological Sciences, University of Maryland Baltimore County, Baltimore, Maryland, USA; University of Southern California

## Abstract

The genome of *Shewanella* sp. strain ISO12, which was isolated from the intestine of wild-caught *Fundulus heteroclitus*, was sequenced and is reported here. Bioinformatic analysis revealed genes encoding the bacteriocin marinocine and those potentially associated with probiotic activity. The genome sequence will assist in further identifying probiotic and other antibacterial processes.

## ANNOUNCEMENT

Application of probiotic bacteria to mitigate microbial pathogen proliferation and to acquire health benefits is a strategy frequently used by the aquaculture industry (1). As part of a study to identify prospective probiotic bacteria, strain ISO12 was isolated from a 24-h enrichment culture grown at 28°C in Trypticase soy broth (Difco) supplemented with 2% NaCl (TSB2) prepared from intestinal extracts of an adult wild-caught *Fundulus heteroclitus* specimen according to IACUC protocol 2013-DM-09-13. Phylogenetic analysis of the 16S rRNA gene (2) identified ISO12 as a *Shewanella* sp. (GenBank accession number KT957943). *Shewanella* spp. have been found to provide improved immunological response (3), growth (4), stress resilience (5), and enhanced disease resistance (3) for aquaculture-raised species (6); they also include pathogenic strains that threaten the health of aquaculture species and their handlers (7, 8). The ISO12 genome sequence will enhance our understanding of the genetic basis for these characteristics.

A single colony was grown in TSB2 overnight at 28°C, and DNA was extracted using the Wizard genomic DNA purification kit (Promega). The genome library was prepared using the Illumina TruSeq Nano DNA library kit and sequenced with an Illumina MiSeq sequencer. The read library contained 15,048,082 paired-end reads with an average length of 245 bp and an average coverage of 49×. After preprocessing and quality control with FastQC (v. 0.11.7) (9), *de novo* assembly by the Pathosystems Resource Integration Center (PATRIC) (v. 3.6.3) (10) using Unicycler (v. 0.4.8) (11) yielded 57 contigs consisting of 4,973,905 bp with an *N*_50_ of 293,228 bp and a GC content of 52.9%. Gene prediction and annotation with the Rapid Annotation using Subsystems Technology (RAST) toolkit (12) indicated 4,654 coding sequences and 98 RNAs. Default parameters were used for all bioinformatic applications, and the publicly available sequence was annotated by the NCBI PGAP.

SEED Viewer (v. 2.0) (12) genome analysis identified *lodA* and *lodB* of the marinocine-producing operon, encoding lysine-epsilon oxidase and a dehydrogenase flavoprotein, respectively. Lysine-epsilon oxidase generates hydrogen peroxide in the presence of lysine (13); the role for *lodB* is unknown but essential for the autolytic and antibiofilm activities of marinocine (14), a bacteriocin produced by several Gram-negative bacteria (15).

The ISO12 genome contains motility, adhesion, and aggregation genes associated with probiotic activity, including flagella (16) and fibronectin (17), as well as bile acid resistance factors, e.g., DamX (18) and cholylglycine hydrolase (19), for protection from the gastrointestinal environment. A 17-gene cluster for mannose-sensitive hemagglutinin may play a role in colonization and adhesion (20). Amylase, lipase, and extracellular serine protease genes important for probiotic activity (21) were also identified.

While ISO12 has not been found to cause disease in fish (22), a hemolysin III homologue, two putative hemolysins, and a pore-forming RTX exotoxin were identified. Genomic and phenotypic studies of ISO12 will expand our understanding of probiosis and pathogenesis in *Shewanella* spp.

### Data availability.

This whole-genome shotgun project has been deposited in DDBJ/ENA/GenBank under the accession number JAAUHW000000000. The version described in this paper is version JAAUHW010000000. Sequence data have been deposited in the Sequence Read Archive under the accession number SRP254567.
